# Circulating Inflammatory, Mitochondrial Dysfunction, and Senescence-Related Markers in Older Adults with Physical Frailty and Sarcopenia: A BIOSPHERE Exploratory Study

**DOI:** 10.3390/ijms232214006

**Published:** 2022-11-13

**Authors:** Anna Picca, Riccardo Calvani, Hélio José Coelho-Júnior, Federico Marini, Francesco Landi, Emanuele Marzetti

**Affiliations:** 1 Fondazione Policlinico Universitario “Agostino Gemelli” IRCCS, 00168 Rome, Italy; 2 Department of Medicine and Surgery, LUM University, 70100 Casamassima, Italy; 3 Department of Geriatrics and Orthopedics, Università Cattolica del Sacro Cuore, 00168 Rome, Italy; 4 Department of Chemistry, Sapienza Università di Roma, 00185 Rome, Italy

**Keywords:** biomarkers, cellular senescence, cytokines, dynapenia, inflammation, multimarker analysis, skeletal muscle, muscle remodeling, physical performance, SASP

## Abstract

Multisystem derangements encompassing musculoskeletal, stress, and metabolic response have been described in older adults with physical frailty and sarcopenia (PF&S). Whether PF&S is also associated with markers of cellular senescence has yet to be explored. To address this research question, we quantified the serum levels of selected inflammatory, mitochondrial, and senescence-associated secretory phenotype (SASP)-related factors in 22 older adults with PF&S (mean age 75.5 ± 4.7 years; 81.8% women) and 27 nonPF&S controls (mean age 75.0 ± 4.4 years; 62.9% women) and evaluated their association with PF&S. Markers of inflammation (interleukin (IL)1-*β*, IL6, and tumor necrosis factor α (TNF-α)), matrix remodeling (Serpin E1, intercellular adhesion molecule 1 (ICAM-1), and tissue inhibitor of metalloproteinases 1 (TIMP-1)), mitochondrial dysfunction (growth/differentiation factor 15 (GDF15) and fibroblast growth factor 21 (FGF21)), Activin A, and glial fibrillary acidic protein (GFAP) were assayed. Serum levels of TNF-α and those of the SASP-related factors ICAM-1 and TIMP-1 were found to be higher, while IL1-*β* and IL6 were lower in PF&S participants compared with controls. Partial least squares discriminant analysis allowed discrimination of PF&S from nonPF&S participants with 74.0 ± 3.4% accuracy. Markers that significantly contributed to the classification were ICAM-1, TIMP-1, TNF-α, GFAP, and IL6. Future studies are warranted to establish whether inflammatory and SASP-related pathways are causally linked to the development and progression of PF&S, and may represent new targets for interventions.

## 1. Introduction

The construct of physical frailty and sarcopenia (PF&S) refers to a pre-disability condition characterized by low physical function and low appendicular lean mass in the absence of mobility disability [1,2]. The defining elements of PF&S are commonly observed in older adults and result from multisystem derangements encompassing musculoskeletal, stress-response, and metabolic systems [3,4]. The efficiency of these systems declines progressively during aging, which may eventually result in a state of compromised function and poor resilience [5,6]. The analysis of modular changes that accompany the transition from “physiological” to “pathological” aging may help disentangle the pathophysiology of PF&S and assist in biomarker and drug target discovery.

Among the pathogenic mechanisms that may contribute to PF&S are the so-called hallmarks of aging. These include several biological processes (i.e., genomic and epigenetic stability, telomere maintenance, nutrient sensing, proteostasis, mitochondrial function, cellular senescence, cell stemness, intercellular signaling, autophagy, microbiome homeostasis, cellular and extracellular mechanical properties, splicing regulation, inflammation) that become impaired during aging [6,7].

Multimarker analytical strategies have been implemented to support the analysis of multisystem derangements and biomarker discovery in the context of frailty [8,9]. Via this approach, patterns of inflammatory, metabolic, and hematologic markers have been found to profile older adults with PF&S [3] and support the tracking of the multifaceted and dynamic nature of this condition [10]. In addition, gut microbial derivatives and mitochondrial components shuttled via extracellular vesicles (i.e., mitochondrial-derived vesicles) have been identified as part of damage-associated molecular patterns in PF&S [11,12]. These mediators have been included among the list of candidate biomarkers of PF&S and are indicated as potential regulators of the gut-muscle axis, possibly through the modulation of inflammation [11,12,13].

Cellular senescence is a state of permanent cell cycle arrest caused by damage-associated signals and is characterized by a sustained release of pro-inflammatory senescence-associated secretory phenotype (SASP) molecules [14]. The accumulation of senescent cells is proposed as a major mechanism driving the aging process and favoring the development of chronic degenerative diseases [15]. However, the relationship between SASP factors and PF&S is yet to be investigated.

To provide an initial appraisal on the subject, in the present study, we measured the serum levels of a panel of mediators related to SASP in a sample of older adults to evaluate their association with PF&S and its defining elements. The panel included pro-inflammatory markers (i.e., interleukin (IL)1-*β*, IL6, and tumor necrosis factor-α (TNF-α)), the growth factor Activin A, the serine proteinase inhibitor Serpin E1, intercellular adhesion molecule 1 (ICAM-1), tissue inhibitor of metalloproteinases 1 (TIMP-1), markers of mitochondrial dysfunction (i.e., growth/differentiation factor 15 (GDF15) and fibroblast growth factor 21 (FGF21)), and the astrocytic intermediate filament glial fibrillary acidic protein (GFAP). Marker selection followed recommendations for geroscience-guided investigations [16], and was based on previous studies showing their implication in pathways and processes relevant to PF&S pathophysiology [3,11,12,17] and senescence-associated secretomes [18].

## 2. Results

### 2.1. Study Population

Forty-nine participants were included in this study, 22 older adults with PF&S (mean age 75.5 ± 4.7 years; 81.8% women) and 27 nonPF&S controls (mean age 75.0 ± 4.4 years; 62.9% women). Demographic, anthropometric, functional, and clinical characteristics of study participants are reported in Table 1. Age, sex distribution, and the number of disease conditions and medications did not differ between groups. Participants with PF&S had higher body mass index (BMI) values, lower short physical performance battery (SPPB) [19] scores, and lower appendicular lean mass (aLM) than non-physically frail, non-sarcopenic (nonPF&S) controls.

### 2.2. Concentrations of Circulating Inflammatory, Mitochondrial, and Senescence Markers According to the Presence of Physical Frailty and Sarcopenia

**Figure 1:**
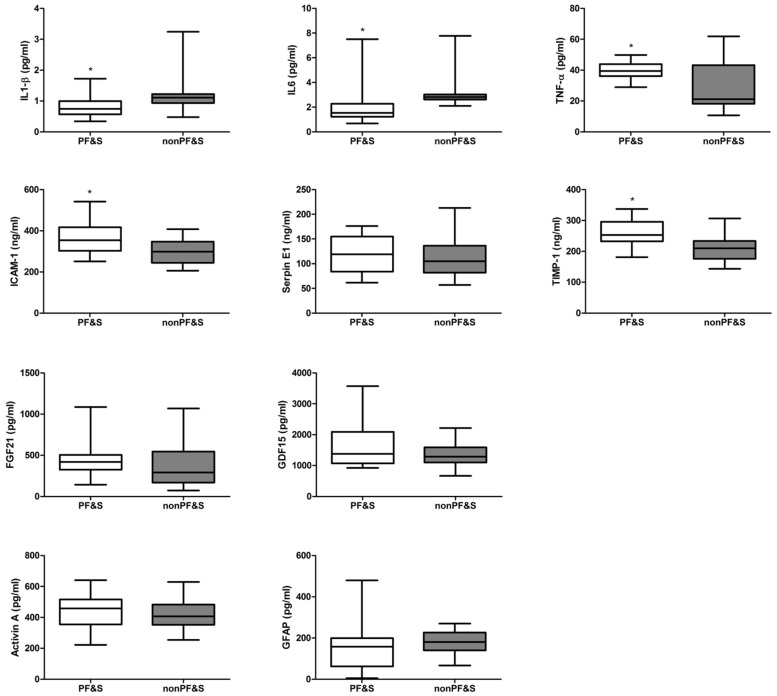
Serum Concentrations of Inflammatory, Mitochondrial, and Senescence-Related Markers in Participants with and without Physical Frailty and Sarcopenia (PF&S). Box plots show the median serum concentration (bold horizontal line), interquartile range (box), and total range of concentrations (whiskers) of markers. Abbreviations: FGF21, fibroblast growth factor 21; GDF15, growth/differentiation factor 15; GFAP, glial fibrillary acidic protein; ICAM-1, intercellular adhesion molecule 1; IL, interleukin; nonPF&S, non-physically frail, non-sarcopenic; TIMP-1, tissue inhibitor matrix metalloproteinase 1; TNF-α, tumor necrosis factor-α; * *p* < 0.05.

Serum levels of the inflammatory markers TNF-α (*p* < 0.05) and those of the SASP-related factors ICAM-1 (*p* < 0.05) and TIMP-1 (*p* < 0.001) were found to be higher, while those of IL1-*β* (*p* < 0.01) and IL6 (*p* < 0.01) were lower in participants with PF&S compared with nonPF&S controls (Figure 1, Table S1).

### 2.3. Correlation Analysis and Hierarchical Clustering of Serum Markers in Participants with Physical Frailty and Sarcopenia and Controls

Results of correlation analyses among circulating inflammatory, mitochondrial, and senescence markers in participants with and without PF&S indicated the existence of specific associations and clustering according to the participant group.

**Figure 2:**
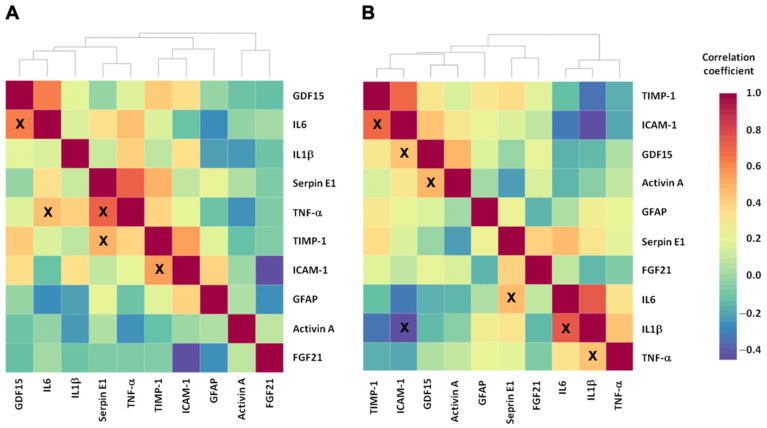
Correlation Matrix and Hierarchical Cluster Analysis of Serum Markers in the Two Participant Groups. Panel (**A**), participants with physical frailty and sarcopenia (PF&S); panel (**B**), participants without PF&S. Abbreviations: FGF21, fibroblast growth factor 21; GDF15, growth/differentiation factor 15; GFAP, glial fibrillary acidic protein; ICAM-1, intercellular adhesion molecule 1; IL, interleukin; TIMP-1, tissue inhibitor matrix metalloproteinase 1; TNF-α, tumor necrosis factor-α; X indicates significant associations (*p* < 0.05).

In those with PF&S, two clusters of mediators were identified, the first including FGF21 and Activin A, and the second including IL1-*β*, IL6, TNF-α, Serpin E1, ICAM-1, TIMP-1, GDF15, and GFAP (Figure 2A). Significant associations were found between IL6 and TNF-α (r = 0.454, *p* = 0.045) and GDF15 (r = 0.64, *p* = 0.006) (Table S2). TNF-α was also associated with Serpin E1 (r = 0.72, *p* = 0.002). Finally, associations were identified between the mediator of matrix remodeling TIMP-1 and ICAM-1 (r = 0.555, *p* = 0.011) and Serpin E1 (r = 0.498, *p* = 0.005).

Two major clusters of associations, different from those identified in participants with PF&S, were found in nonPF&S controls (Figure 2B). One cluster included mediators involved in muscle remodeling, such as the inhibitor of metalloproteases, intercellular adhesion molecules, and markers of mitochondrial dysfunction (i.e., ICAM-1, TIMP-1, Activin A, GDF15, GFAP, Serpin E1, and FGF21). The other cluster included inflammatory markers (i.e., TNF-α, IL1-*β*, and IL6). Significant associations were identified for IL1-*β* with IL6 (r = 0.746, *p* < 0.001), TNF-α (r = 0.437, *p* = 0.026), and ICAM-1 (r = −0.746, *p* = 0.019) (Table S2). IL6 was also significantly associated with Serpin E1 (r = −0.464, *p* = 0.023), while ICAM-1 was associated with TIMP-1 (r = 0.693, *p* < 0.001) and GDF15 (r = 0.436, *p* = 0.026). Finally, Activin A was associated with GDF15 (r = 0.483, *p* = 0.009).

### 2.4. Multivariate Classification and Regression Analyses of Serum Markers in Participants with Physical Frailty and Sarcopenia and Controls

A multivariate classification approach based on partial least squares discriminant analysis (PLS–DA) was used to verify whether a statistically significant difference existed in the profile of inflammatory, mitochondrial dysfunction, and senescence-related factors between PF&S and nonPF&S participants. Results were evaluated through a repeated double cross-validation (DCV) procedure with 10 and eight cancelation groups in the outer and inner loops, respectively. On average, the model included three latent variables and yielded an overall classification accuracy of 70.4 ± 3.1%. The model allowed for correct classification of 71.0 ± 4.2% PF&S participants and 69.6 ± 3.9% nonPF&S controls. Inspection of the contribution of individual experimental variables to the classification model indicated that five out of the 10 measured markers significantly contributed to the discrimination: ICAM-1, TIMP-1, TNF-α (on average higher for PF&S), GFAP, and IL6 (on average higher for controls).

**Figure 3:**
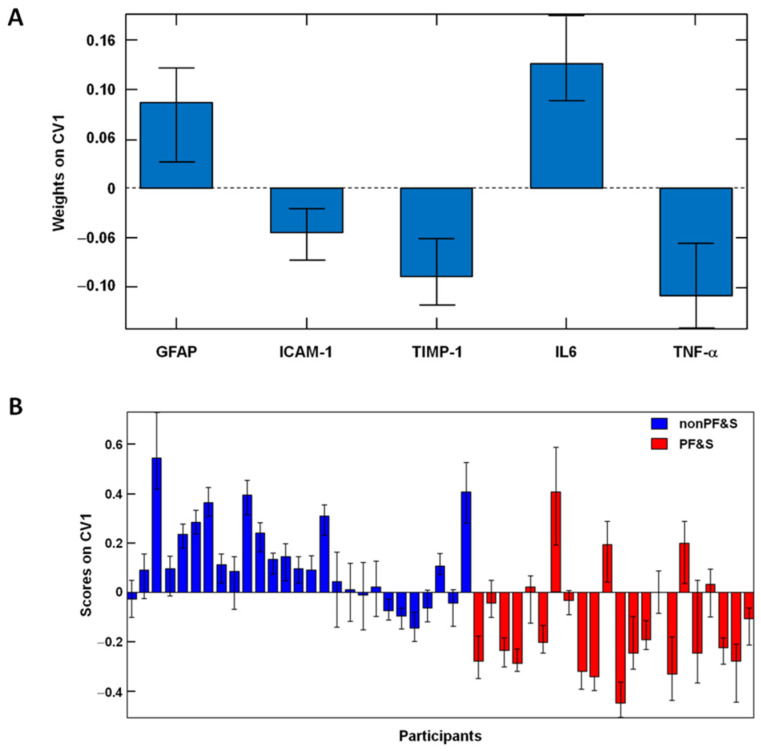
Results of Partial Least Squares Discriminant Analysis on the Reduced Subset of Relevant Circulating Markers. (**A**) Variable weights along the only canonical variate (CV) of the model. Results indicate that all the five selected markers significantly contribute to the discrimination between participants with physical frailty and sarcopenia (PF&S) and nonPF&S controls. (**B**) Double cross-validation scores with 95% confidence intervals of outer loop samples along the only canonical variate of the model. Abbreviations: GFAP, glial fibrillary acidic protein; ICAM-1, intercellular adhesion molecule 1; IL, interleukin; TIMP-1, tissue inhibitor matrix metalloproteinase 1; TNF-α, tumor necrosis factor-α.

An additional classification model was built on the reduced subset of relevant circulating markers (Figure 3A). The model provided an overall 74.0 ± 3.4% classification accuracy on the outer loop samples (75.0 ± 4.9% for PF&S; 72.8 ± 4.7% for nonPF&S). The good discrimination between the two participant groups can be appreciated graphically in Figure 3B, where the projection of outer DCV loop samples onto the only canonical variate of the model is depicted. The figure allows for appreciating that most PF&S participants fall at negative values of the component, while most nonPF&S participants have positive scores.

**Figure 4:**
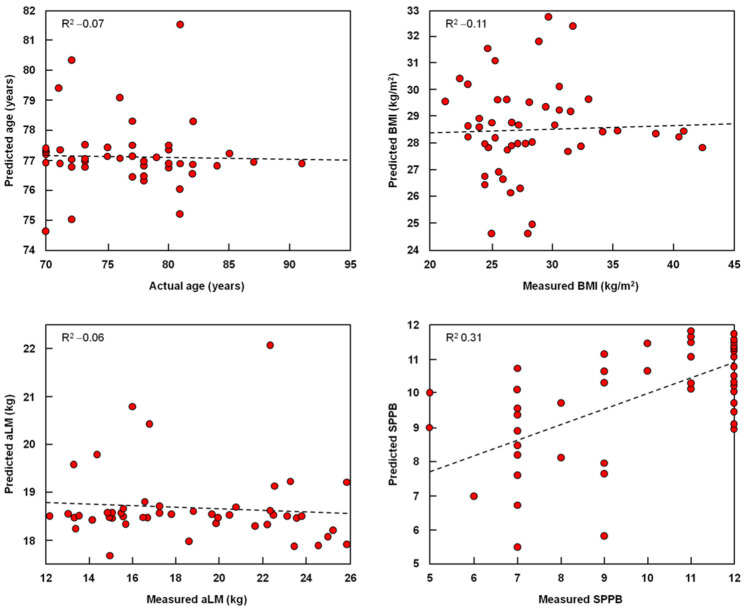
Kernel Partial Least Squares Models to Explore Relationships between Circulating Markers and Age, Anthropometric Measures, and Physical Performance. The models compared the actual values of individual variables and model predictions in the outer double cross-validation loop. Abbreviations: aLM, appendicular lean mass; BMI, body mass index; SPPB, short physical performance battery.

Finally, multivariate regression models using kernel PLS were built to explore the relationship between circulating markers and participant age, BMI, aLM, and SPPB scores (Figure 4). Models were validated by DCV, with 10 and eight cancelation groups in the outer and inner loops, respectively. Models for the prediction of age, BMI, and aLM had R^2^ values close to 0, indicating the absence of a significant relationship between the analyzed variables. A higher R^2^ value (0.31), though still relatively low, was obtained for the model relating the circulating markers to SPPB scores.

## 3. Discussion

Perturbations of inflammatory and/or metabolic pathways have been described in older adults with PF&S with specific patterns of associations [3,11,12,17,20]. Most of those perturbations may be attributed to the chronic state of low-grade inflammation observed during aging, called inflamm-aging, which conveys pro-sarcopenic and pro-disability effects [21,22,23,24].

In the present study, we sought to gather additional information on the inflammatory changes observed in older adults with PF&S and evaluate whether these alterations are related to senescence signaling and are, therefore, part of SASP. To this aim, we built an analytic panel of mediators including the senescence-related factors Activin A, Serpin E1, ICAM-1, and TIMP-1, the markers of mitochondrial dysfunction GDF15 and FGF21, and the astrocytic intermediate filament GFAP, in association with the pro-inflammatory mediators IL1-*β*, IL6, and TNF-α. The approach for biomarker selection was consistent with recommendations for geroscience-guided studies [16]. Individual markers were chosen based on previous studies indicating their relevance to PF&S pathophysiology [3,11,12,17] and senescence-associated secretomes [18]. The panel of mediators was measured in serum samples obtained from older adults with and without PF&S, recruited in the BIOmarkers associated with Sarcopenia and PHysical frailty in EldeRly pErsons (BIOSPHERE) study [25].

Our analyses revealed a differential cytokine pattern in older adults with PF&S compared with the nonPF&S group. This finding is in line with the notion of cytokine network dysregulation as a converging point of mechanistic pillars of aging and a major driver of age-associated conditions [23,26,27,28]. Inflamm-aging is sustained by the release of several cytokines, including IL1-*β*, IL6, and TNF-α, by local inflammatory cells (e.g., neutrophils and macrophages) [23]. This occurs in response to a plethora of stimuli (e.g., pathogens, gut microbial products, endogenous damage-associated molecular patterns) [23]. From this perspective, the pattern of inflammatory mediators in older adults with PF&S may represent an endophenotypic expression of inflamm-aging, although it may not be limited to this. TNF-α is a pro-inflammatory cytokine released by diseased tissues. TNF-α induces skeletal muscle atrophy through both direct and indirect mechanisms [29] and depresses muscle contractility in vitro and in vivo [30,31]. Studies in animal models and humans showed that higher circulating levels of TNF-α and IL6 were associated with reduced muscle mass and strength [32,33]. These findings suggest that a composite measure of the two cytokines may be better suited than either cytokine alone for assessing the inflammatory status in the context of frailty and sarcopenia [34]. Our results further highlight the existence of a relationship between TNF-α and IL6 in older adults with PF&S. However, IL6 levels were reduced in participants with PF&S compared with nonPF&S controls. This counterintuitive finding may be explained by the “double-edged sword” nature of IL6, which can either promote muscle anabolism or induce muscle catabolism depending on the tissular *milieu* [35]. Moreover, recent evidence indicates that circulating IL6 is associated with frailty and sarcopenia in people younger than 75, but not in those ≥75 [10]. Further studies are needed to better characterize the context- and age-dependent effects of the TNF-α/IL6 dyad on muscle homeostasis in advanced age.

Higher circulating levels of TIMP-1 and ICAM-1 were found in participants with PF&S relative to controls, which may be indicative of perturbations in cellular senescence pathways. Extracellular matrix (ECM) remodeling has emerged as a critical regulator of tissue homeostasis during aging [36]. The combined actions of TIMPs and matrix metalloproteinases (MMPs) regulate the composition and mechanical properties of ECM [36]. An imbalance between MMPs and TIMPs activities alters stem cell behavior through reducing the availability of ECM-bound growth factors, and induces the development of a senescent phenotype [36,37]. Perturbations in ECM dynamics may also promote tissue fibrosis. As observed in animal models of renal fibrosis, several inflammatory mediators, including ICAM-1, are substrate of TIMPs and induce tissue inflammation and damage upon TIMP-1 stimulation [38]. The positive association between serum levels of Serpin E1, TNF-α, and TIMP-1 in older adults with PF&S suggests a role for Serpin E1 and TIMP-1 in buffering the pro-atrophy muscle ECM remodeling as part of the senescence-related response. Serpin E1, also known as plasminogen activator inhibitor-1 (PAI-1) due to its role as a master regulator of the plasminogen system, has recently been identified in muscle where it seems to be involved in tissue remodeling [39]. Serpin E1 is an upstream inhibitor of plasmin activation and modulates the activity of MMPs by reducing the activation of pro-MMPs [40]. As a result of this regulation, Serpin E1 plays a central role in ECM remodeling. Plasmin can also trigger MMP secretion, whereas its zymogen (i.e., plasminogen) regulates PAI-1 secretion [40]. Therefore, upregulation of PAI-1 may serve as a negative feedback mechanism aimed at limiting plasmin- and MMP-driven ECM degradation [40]. Together with PAI-1 secretion, high levels of TIMPs have been documented [41,42,43]. For instance, in the setting of fibrotic signaling cascades, a concomitant increase in the levels of PAI-1 and TIMPs has been reported [41,42,43]. In this context, the activity of TIMPs, by exerting a direct inhibitory effect on MMPs, may result in blockade of ECM degradation.

GFAP is the main intermediate filament protein of astrocytes [44]. Following brain injury, GFAP and its breakdown products are rapidly released into biofluids [45]. Increased levels of GFAP in the cerebrospinal fluid have been detected in people with neurodegenerative diseases, including Alzheimer’s disease and Parkinson’s disease [46]. Higher circulating GFAP concentrations have also been associated with faster cognitive decline [47] and a higher risk of dementia [48,49]. The reduced levels of GFAP found in participants with PF&S seem to be at odds with existing evidence. However, GFAP expression in astrocytes may increase with age also in healthy individuals [50]. Therefore, the relationship between circulating GFAP levels and age-related conditions needs to be further explored.

Although reporting novel findings, our study has limitations that deserve discussion. Due to the small sample size and the single-center nature of the study, results should be considered preliminary. However, given the clinical relevance of the PF&S construct [2], our findings may serve as the foundation for larger investigations on the subject. Furthermore, PLS–DA is particularly suited for analyzing matrices in which the number of variables is larger than the number of participants, variables are correlated with each other, and differences in biological factors are heterogeneous across individuals [51]. The DCV procedure confirmed the reliability of PLS–DA models [52]. Physical activity and nutritional habits, as well as the use of specific drug classes (e.g., anti-inflammatory drugs) may have influenced circulating levels of the assayed biomolecules [53,54,55]. None of the participants reported regular use of anti-inflammatory drugs. However, physical activity levels and diet composition were not recorded. The investigation followed a cross-sectional design, which does not allow mechanistic and temporal relationships to be established between circulating markers and PF&S development or progression. Finally, although we assayed a consistent number of biomolecules, we could not analyze all inflammatory and cellular senescence markers that are possibly involved in PF&S. Hence, the existence of additional and more powerful geroscience biomarkers of PF&S cannot be excluded.

## 4. Materials and Methods

### 4.1. Study Participants

Participants were recruited as part of BIOSPHERE, a cross-sectional, case-control study, aimed at identifying and validating a panel of biomarkers for PF&S through a multimarker approach [25]. Details about the study protocol, inclusion and exclusion criteria, and procedures adopted for participant recruitment have been reported elsewhere [17,56]. The main study enrolled 200 community-dwelling older adults (i.e., 70+ years old), 100 with PF&S and 100 nonPF&S controls. Based on the availability of serum samples, 49 participants were included in the present study, 22 with PF&S and 27 nonPF&S controls. Demographic, functional, body composition, and clinical characteristics of this subset were not significantly different from the remaining BIOSPHERE participants. PF&S was diagnosed using the operational definition elaborated within the Sarcopenia and Physical fRailty IN older people: multi-componenT Treatment strategies (SPRINTT) project [57,58]. More specifically, participants were included in the PF&S group if showing (a) low physical performance (i.e., SPPB [18] score between 3 and 9); (b) low absolute or BMI-adjusted aLM as measured by dual X-ray absorptiometry, according to the cut-points of the Foundation for the National Institutes of Health (FNIH) sarcopenia project [59]; and (c) absence of major mobility disability (i.e., retained ability to complete a 400-m walk test) [60]. Control participants had SPPB scores of 10 to 12 and aLM values above the FNIH cut-points. The study protocol was approved by the Ethics Committee of the Università Cattolica del Sacro Cuore (Rome, Italy; protocol number: 8498/15), and all procedures were performed in accordance with the ethical standards laid down in the 1964 Declaration of Helsinki and its later amendments. All participants provided written informed consent prior to inclusion.

### 4.2. Measurement of Inflammatory, Mitochondrial, and Senescence-Related Markers in Serum

Serum was collected after overnight fasting as previously described [3] and used to measure a panel of 10 mediators. Biomolecules were selected based on their involvement in pathways and processes related to senescence and PF&S pathophysiology [3,11,61]. IL1-*β*, IL6, and TNF-α were quantified simultaneously as part of the 27 Bio-Plex Pro Human Cytokine 27-plex Assay (#M500KCAF0Y, Bio-Rad Laboratories Inc., Hercules, CA, USA), a magnetic bead-based immunoassay run on a Bio-Plex^®^ System with Luminex xMAP^®^ Technology (Bio-Rad). Activin A was measured using a commercially available kit (Human/Mouse/Rat Activin A Quantikine ELISA Kit #DAC00B, R&D System, Minneapolis, MN, USA), while FGF21, GDF15, GFAP, ICAM-1, Serpin E1, and TIMP-1 were assayed using commercially available kits on an ELLA™ automated immunoassay system (Bio-Techne, San Jose, CA, USA).

### 4.3. Statistical Analysis

The Kolmogorov–Smirnov test was used to verify the normal distribution of data. Data of normally distributed variables are shown as means ± standard deviations, while those with non-normal distribution are reported as medians and interquartile range (IQR). In case of normal distribution, differences in continuous variables between PF&S and nonPF&S groups were determined via independent sample *t*-test. Otherwise, the non-parametric test Mann–Whitney U was used. Differences in categorical variables between groups were determined via χ^2^ statistics. Correlations between circulating markers were explored in the two participant groups separately by Pearson’s correlation tests. All tests were two-sided, with statistical significance set at *p* < 0.05, and were performed using the GraphPrism 5.03 software (GraphPad Software, Inc., San Diego, CA, USA). Hierarchical cluster analysis was run according to the Ward’s minimum variance method to visually inspect relationships among all assayed markers in the two participant groups. The analysis was conducted using the freely available R software (RStudio, PBC, Boston, MA, USA). Cluster heatmaps based on Pearson’s correlation coefficients were used as the visualization method.

A multivariate classification approach based on PLS–DA was used to verify whether a statistically significant difference existed in the profile of circulating markers between PF&S and nonPF&S participants. PLS–DA is a classification method that exploits the advantages of the PLS algorithm for dealing with correlated variables and experimental conditions, where predictors are more numerous than training samples [51]. The PLS algorithm was originally developed for regression problems. It relies on the projection of the predictor matrix X onto a reduced space of orthogonal latent variables, yielding a matrix of scores T (coordinates of the samples onto the latent variables subspace):

(1) T=XR

where R is a matrix of weights determining the projection.

A regression model is then established between the scores T and the response y to be predicted:

(2) y=Tq

where q is the regression coefficient.

The same approach can be used for classification by using a dummy binary y coding for class-belonging: the elements of y can be either 1 if the sample belongs to a category (here PF&S) or 0 if it belongs to the other (here, nonPF&S). In this case, since the model predictions are not binary but real-valued, classification is achieved by applying linear discriminant analysis on the vector of predicted responses.

To evaluate whether the multivariate profile of circulating markers could be related to any relevant clinical data, a non-linear version of the PLS algorithm (i.e., KPLS) was adopted. In KPLS, the scores in Equation (1) are not extracted directly from the experimental data X, but are derived from a non-linear projection of the data, implicitly achieved by kernel transformation (here, using a Gaussian kernel) [62].

In both cases, model validation was achieved through DCV [52]. DCV consists of two loops of cross-validation nested in one another: the outer loop mimics an external test set, while the inner loop is used for model selection (i.e., choosing the optimal number of latent variables or the best width of the Gaussian kernel). The procedure was repeated 50 times, changing the distribution of the samples in the different cancelation groups, which made it possible to calculate confidence intervals and figures of merit for all model parameters. Analyses were performed using in-house routines running under MATLAB R2015b environment (The MathWorks, Natick, MA, USA).

## 5. Conclusions

Findings from our investigation indicate that a specific pattern of circulating inflammatory and senescence-related markers characterizes older adults with PF&S. Whether cellular senescence and SASP are causally related to the development and progression of PF&S and may therefore represent new targets for intervention warrants investigation.

## Figures and Tables

**Table 1:** Main characteristics of study participants according to the presence of physical frailty and sarcopenia (PF&S).

	PF&S (n = 22)	nonPF&S (n = 27)	*p*
Age, years (mean ± SD)	75.5 ± 4.7	75.0 ± 4.4	0.719
Gender (female), n (%)	18 (81.8)	17 (62.9)	0.146
BMI, kg/m^2^ (mean ± SD)	29.9 ± 4.4	26.6 ± 2.2	0.010
SPPB (mean ± SD)	7.8 ± 1.6	11.3 ± 0.9	<0.001
aLM, kg (mean ± SD)	16.3 ± 3.6	20.3 ± 4.0	<0.001
aLM_BMI_ (mean ± SD)	0.55 ± 0.12	0.86 ± 0.28	<0.001
Number of disease conditions * (mean ± SD)	2.5 ± 1.6	1.8 ± 1.5	0.121
Number of medications ** (mean ± SD)	3.3 ± 2.0	2.9 ± 1.9	0.477

Abbreviations: aLM, appendicular lean mass; BMI, body mass index; nonPF&S, non-physically frail, non-sarcopenic; SD, standard deviation; SPPB, short physical performance battery. * Includes hypertension, coronary artery disease, prior stroke, peripheral vascular disease, diabetes, chronic obstructive pulmonary disease, and osteoarthritis. ** Includes prescription and over-the-counter medications.

## Data Availability

The data presented in this study are available on request from the corresponding author.

## Supplementary Materials

The following supporting information can be downloaded at: https://www.mdpi.com/article/10.3390/ijms232214006/s1.
