# Supplementary material for: Circulating Inflammatory, Mitochondrial Dysfunction, and Senescence-Related Markers in Older Adults with Physical Frailty and Sarcopenia: A BIOSPHERE Exploratory Study
Source: Int J Mol Sci. 2022 Nov 13;23(22):14006. doi: 10.3390/ijms232214006 (PMC9692456; doi:10.3390/ijms232214006)
Supplement: Supplementary file 1 [file ijms-23-14006-s001.zip › ijms-1917218-supplementary.pdf]

Table S1. Serum Concentrations of Biomarkers According to the Presence of Physical Frailty and Sarcopenia (PF&S).

	**PF&S (n=22)**	**nonPF&S (n=27)**	**P**
IL1-β (pg/mL) (mean ± SD)	0.811 ± 0.325	1.2 ± 0.553	0.008
IL6 (pg/mL) (mean ± SD)	1.9 ± 1.44	3.08 ± 1.2	0.004
TNF-α (pg/mL) (median, IQR)	39.4 (7.34)	21.1 (23.3)	0.010
Activin A (pg/mL) (mean ± SD)	435 ± 107	415 ± 87.0	0.471
ICAM-1 (pg/mL) (mean ± SD)	362060 ± 77143	299826 ± 57260	0.002
Serpin E1 (pg/mL) (mean ± SD)	120567 ± 36221	110759 ± 37346	0.402
TIMP-1 (pg/mL) (mean ± SD)	256226 ± 43488	211602 ± 42576	<0.001
GFAP (pg/mL) (mean ± SD)	151 ± 106	180 ± 54.6	0.23
GDF15 (pg/mL) (median, IQR)	1377 (945)	1284 (476)	0.24
FGF21 (pg/mL) (mean ± SD)	432 ± 207	372 ± 254	0.385

Mean ± standard deviation and median values (interquartile range) are reported for normal and non-normal distributed variables, respectively. *Abbreviations*: FGF21, fibroblast growth factor 21; GDF15, growth/differentiation factor 15; GFAP, glial fibrillary acidic protein; ICAM-1, intercellular adhesion molecule 1; IL, interleukin; IQR, interquartile range; SD, standard deviation; TIMP-1, tissue inhibitor matrix metalloproteinase 1; TNF-α, tumor necrosis factor-α.

Table S2. Correlation Analysis of Serum Markers of Inflammation, Mitochondrial Dysfunction, and Senescence in Participants with Physical Frailty and Sarcopenia (PF&S) and Non Physically Frail, Non Sarcopenic (nonPF&S) Controls.

					**PF&S (n=22)**					
	**IL1-β**	**IL6**	**TNF-α**	**ICAM-1**	**Serpin E1**	**TIMP-1**	**Activin A**	**GFAP**	**GDF15**	**FGF21**
**IL1-β**	—									
	—									
**IL6**	r=0.160	—								
	P=0.500	—								
**TNF-α**	r=0.401	r=0.454	—							
	P=0.079	P=0.045	—							
**ICAM-1**	r=0.349	r=–0.126	r=0.191	—						
	P=0.143	P=0.608	P=0.433	—						
**Serpin E1**	r=0.110	r=0.343	r=0.720	r=0.09	—					
	P=0.696	P=0.211	P=0.002	P=0.740	—					
**TIMP-1**	r=0.083	r=0.157	r=0.386	r=0.555	r=0.498	—				
	P=0.729	P=0.507	P=0.093	P=0.011	P=0.050	—				
**Activin A**	r=–0.245	r=–0.034	r=–0.265	r=–0.007	r=0.046	r=–0.133	—			
	P=0.299	P=0.886	P=0.259	P=0.978	P=0.865	P=0.555	—			
**GFAP**	r=–0.229	r=–0.278	r=–0.118	r=0.394	r=0.205	r=0.177	r=0.042	—		
	P=0.346	P=0.250	P=0.631	P=0.086	P=0.446	P=0.457	P=0.859	—		
**GDF15**	r=0.182	r=0.640	r=0.169	r=0.360	r=–0.021	r=0.423	r=–0.120	r=–0.024	—	
	P=0.484	P=0.006	P=0.516	P=0.143	P=0.938	P=0.080	P=0.637	P=0.923	—	
**FGF21**	r=–0.117	r=0.014	r=–0.075	r=–0.455	r=–0.079	r=–0.071	r=0.084	r=–0.271	r=–0.136	—
	P=0.624	P=0.953	P=0.753	P=0.051	P=0.780	P=0.759	P=0.717	P=0.261	P=0.604	—
					**nonPF&S (n=27)**					
**IL1-β**	—									
	—									

**IL6**	r=0.746	—								
	P<0.001	—								
**TNF-α**	r=0.437	r=0.319	—							
	P=0.026	P=0.112	—							
**ICAM-1**	r=–0.475	r=–0.350	r=–0.223	—						
	P=0.019	P=0.093	P=0.296	—						
**Serpin E1**	r=0.308	r=0.464	r=0.174	r=0.145	—					
	P=0.143	P=0.023	P=0.416	P=0.461	—					
**TIMP-1**	r=–0.371	r=–0.150	r=–0.202	r=0.693	r=0.355	—				
	P=0.062	P=0.465	P=0.322	P<0.001	P=0.064	—				
**Activin A**	r=–0.072	r=–0.181	r=0.080	r=0.286	r=–0.250	r=0.137	—			
	P=0.726	P=0.376	P=0.699	P=0.140	P=0.199	P=0.470	—			
**GFAP**	r=0.240	r=0.024	r=0.199	r=0.206	r=0.223	r=0.283	r=–0.008	—		
	P=0.259	P=0.910	P=0.351	P=0.323	P=0.284	P=0.153	P=0.968	—		
**GDF15**	r=–0.168	r=–0.196	r=0.03	r=0.436	r=–0.032	r=0.294	r=0.483	r=0.150	—	
	P=0.434	P=0.359	P=0.890	P=0.026	P=0.878	P=0.129	P=0.009	P=0.474	—	
**FGF21**	r=–0.171	r=0.075	r=–0.091	r=0.072	r=0.390	r=0.165	r=0.138	r=–0.183	r=0.194	—
	P=0.403	P=0.716	P=0.658	P=0.737	P=0.060	P=0.421	P=0.501	P=0.393	P=0.364	—

Abbreviations: FGF21, fibroblast growth factor 21; GDF15, growth/differentiation factor 15; GFAP, glial fibrillary acidic protein; ICAM-1, intercellular adhesion molecule 1; IL, interleukin; TIMP-1, tissue inhibitor matrix metalloproteinase 1; TNF-α, tumor necrosis factor-α.
